# Real-Time Prediction of Resident ADL Using Edge-Based Time-Series Ambient Sound Recognition

**DOI:** 10.3390/s24196435

**Published:** 2024-10-04

**Authors:** Cheolhwan Lee, Ah Hyun Yuh, Soon Ju Kang

**Affiliations:** School of Electronic and Electrical Engineering, College of IT Engineering, Kyungpook National University, 80 Daehakro, Bukgu, Daegu 41566, Republic of Korea; chlee4831@knu.ac.kr (C.L.); 0828lucy@gmail.com (A.H.Y.)

**Keywords:** internet of medical things, edge AI, human activity recognition, sound classification, ambient assisted living

## Abstract

To create an effective Ambient Assisted Living (AAL) system that supports the daily activities of patients or the elderly, it is crucial to accurately detect and differentiate user actions to determine the necessary assistance. Traditional intrusive methods, such as wearable or object-attached devices, can interfere with the natural behavior of patients and may lead to resistance. Furthermore, non-intrusive systems that rely on video or sound data processed by servers or the cloud can generate excessive data traffic and raise concerns about the security of personal information. In this study, we developed an edge-based real-time system for detecting Activities of Daily Living (ADL) using ambient noise. Additionally, we introduced an online post-processing method to enhance classification performance and extract activity events from noisy sound in resource-constrained environments. The system, tested with data collected in a living space, achieved high accuracy in classifying ADL-related behaviors in continuous events and successfully generated user activity logs from time-series sound data, enabling further analyses such as ADL assessments. Future work will focus on enhancing detection accuracy and expanding the range of detectable behaviors by integrating the activity logs generated in this study with additional data sources beyond sound.

## 1. Introduction

The increasing average life expectancy and the diversification of family structures have led to a significant rise in the number of elderly individuals living alone. Consequently, elderly care, particularly for individuals with conditions like dementia who face challenges in daily living, has become a critical concern. Traditionally, these challenges have been managed through facilities and human caregivers. However, as the population in need of assistance rapidly grows, this approach may not be sustainable in the long term.

To address these issues, research into Ambient Assisted Living (AAL) systems is advancing. AAL systems use intelligent objects to assist individuals with daily activities [1,2,3,4]. The success of AAL relies on technology capable of detecting and classifying user actions by observing behaviors through smart objects integrated into the living environment.

Activities of Daily Living (ADL) are a standard metric used to evaluate an individual’s ability to perform routine tasks, such as eating, maintaining hygiene, and moving around, and to identify areas where assistance may be needed. ADL assessments are also used as diagnostic tools for tracking cognitive disorders like dementia [5,6]. Traditionally, ADL measurements have been gathered through surveys completed by patients or caregivers. However, this method is often subjective and prone to inaccuracies due to memory reliance. Therefore, efforts are underway to develop technological solutions for more objective ADL detection [7,8,9].

IoT systems designed to recognize ADL frequently use wearable devices or sensors attached to objects to gather data through direct interaction with users [4,10,11]. While this intrusive approach can provide accurate behavioral data, ensuring consistent device usage among elderly individuals is challenging. Moreover, these devices may interfere with natural behavior, leading to discomfort and resistance. The specialized nature of such systems also results in configuration challenges and scalability limitations.

In contrast, non-intrusive systems estimate behaviors indirectly from user actions, keeping devices hidden and allowing users to maintain their routines with minimal disruption [7,8,12]. These systems are more attractive for long-term monitoring as they avoid the discomfort and inconsistency associated with wearable devices. However, analyzing time-series data such as sound with deep learning models often results in fragmented and error-prone inferences, requiring post-processing techniques to correct these issues and produce reliable activity logs.

Typically, deep learning models send data to servers or the cloud for processing, which raises concerns about privacy and generates significant network traffic, particularly when sensitive information like health data or conversations is transmitted [13]. To mitigate these concerns, there is a growing trend towards edge-based systems, where data are processed locally, reducing privacy risks and minimizing network traffic while maintaining real-time performance.

Handling time-series data in edge computing environments presents unique challenges. Edge devices are typically resource-constrained, with limited processing power and memory compared to centralized servers. Time-series data, such as ambient noise, require continuous real-time processing, which can overwhelm edge devices if not efficiently optimized. Furthermore, the nature of time-series data makes them prone to fragmentation, especially in environments with overlapping sounds and background noise. This can result in inaccurate or incomplete inferences, complicating the classification of ADL-related events. Therefore, robust techniques are needed to manage this fragmentation and ensure efficient data processing within the limited computational resources of edge devices.

Additionally, edge-based processing requires real-time inference, meaning that latency must be minimized without sacrificing accuracy. The need for continuous data processing in noisy environments creates a significant trade-off between low latency and reliable classification. Achieving this balance involves designing models and algorithms that operate efficiently within the constraints of edge hardware while maintaining high accuracy.

In this study, we propose a real-time ADL analysis system that utilizes ambient noise in an edge computing environment. We also introduce an online post-processing method to organize fragmented inference results and convert them into ADL events. The system comprises edge nodes installed in individual spaces, along with a hub device that manages the nodes and collects data. Each edge node performs data collection, inference, and post-processing locally, transmitting only event information to the hub device, enabling high-level analysis of behavioral data from the entire house.

The main contributions of this study are as follows:

1. Development of an easy-to-install, edge-based, non-intrusive real-time behavior detection system for seamless integration into existing living spaces.
2. Proposal of post-processing techniques to improve inference accuracy and abstract results for high-level analysis in resource-constrained environments.

## 2. Related Works

### 2.1. Human Activity Recognition

Research aimed at measuring human behavior through technology is being conducted for various purposes as in Table 1, such as healthcare and elderly care [2,14]. These approaches can generally be divided into two categories: methods that use sensor data from devices carried by individuals, such as wearables or smartphones, and methods that install multiple sensors in an environment to analyze the data collected from that space. More complex methods may involve analyzing video or audio data.

Wearable devices or smartphones typically use accelerometer data to directly track human movement [15,16,28] or physiological data such as PPG (photoplethysmography) and ECG (electrocardiogram) [18,19,29]. Smartphones can also leverage built-in sensors such as WiFi signal strength, temperature, and magnetometers. In the realm of smart home systems, research often combines motion sensors with environmental data or directly interacts with objects equipped with measurement devices [10,21]. Some studies also use environmental data alongside sensor data to observe behavior through changes in factors like air quality resulting from user actions [7,11,30].

Video-based methods have been widely researched for behavior analysis using RGB video data [22,31]. However, privacy concerns limit their suitability for home environments. As a result, alternative methods using ambiguous video formats such as LiDAR or thermal imaging are being explored [12,23]. Studies that utilize sound, particularly in environmental sound classification, are advantageous for non-intrusive systems. Sound can capture information from an entire space regardless of sensor orientation, making it ideal for constructing non-intrusive systems [8,24,32].

### 2.2. Time-Series Ambient Sound Classification with Edge AI Technologies

Unlike voice data, environmental sound data lack structured features like phonemes or distinct frequency bands [33]. This makes it difficult to apply many of the signal processing techniques typically used for speech. Environmental sound data have numerous applications, from detecting outdoor events to identifying indoor behaviors or anomalies. Datasets such as UrbanSound8k [34], AudioSet [35], and DESED (Domestic Environment Sound Event Detection Dataset) [36] encompass a wide variety of environmental sounds. Research using deep learning models on these datasets is ongoing [25,37,38]. While these datasets are useful for developing models, real-world environments often introduce additional noises that are not accounted for in the datasets. Thus, it is essential to develop methods to handle these extraneous noises effectively.

As AI technology progresses, efforts are being made to implement it on devices like smartphones, Raspberry Pi, and Microcontroller Units (MCUs). New MCUs with higher clock speeds and greater memory are being developed to support deep learning models, which typically require substantial memory and matrix operations [39]. Unlike server-based systems, edge devices have limited resources and are closely connected to their physical environment. Research is focusing on optimizing models to operate efficiently in edge environments with minimal resource consumption [40]. There is also ongoing research into extracting features efficiently from sensor data [25,26].

Additionally, these advancements enable the development of new services. For example, previously complex data processing models are now feasible on edge devices [27,41]. Furthermore, time-series sensor data, often handled by embedded systems, are also being explored for use in edge computing applications [17,20].

## 3. Methodology

In this study, we aimed to develop an edge-based system capable of real-time detection of human actions by utilizing noise data generated by people’s activities in daily living environments. The issues that needed to be addressed in implementing the proposed system are as follows:

1. Real-Time Processing of Time-Series Sound Signals
  It should be capable of processing and inferring time-series sound signals in real time. To achieve this, we designed an edge device with a hybrid architecture consisting of low-power cores and high-performance cores, and we developed and implemented software architecture to effectively utilize it. Additionally, we implemented an AI model architecture suitable for use on edge devices and applied optimization methods such as quantization to enable real-time operation with low resource consumption.
2. Ability to Identify Relevant Sounds in Overlapping Noise
  The system must be able to detect sounds related to the actions of interest in environments where various noises and meaningful data are overlapping. In this study, we built a dataset for model training using sound data collected directly from the actual environment where the system is to be installed. Furthermore, by applying a post-processing technique using voting, we improved the accuracy for continuous sound data and rectified inference results where complex signals were mixed, enabling us to identify meaningful signals.
3. Generation of Outputs Integrable with Other Data
  The detected action events must be produced in an output format that allows integration with other data. We interpreted the action data identified through post-processing according to the installation environment and generated action event logs.

## 4. System Overview

We aimed to establish a system that should meet the following criteria:

1. The installation and management should be simple, with minimal device configuration.
2. All data processing should occur within the edge device, without the need for external data transmission.
3. The system should be capable of operating in real time, even under exceptional circumstances.

**Figure 1:**
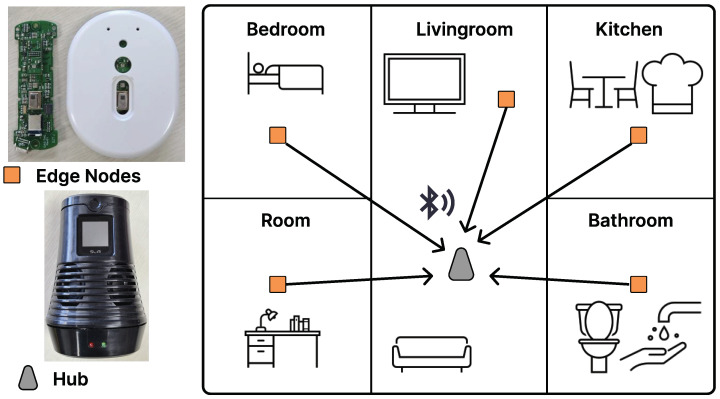
System overview.

The proposed system consists of several edge node devices and a hub device, as shown in Figure 1. Each edge node is installed in a designated space and collects data within its coverage area, indicating the location of event occurrences. The hub device connects to all edge nodes, enabling it to monitor time synchronization, battery levels, and the connection status for each node. When a sound exceeding a certain threshold is detected, the edge node samples the sound, extracts features, performs inference and post-processing to generate an event log, and transmits it to the hub. The hub stores these event logs with timestamps and locations, allowing for high-level analysis, such as examining interactions between behaviors across multiple spaces. If needed, the hub can also transmit data externally.

In addition, the system functions as a platform for collecting sound data related to activities, an essential step in building the foundational model. It gathers sound data tied to behavior, along with corresponding location and timestamp information. These data can later be compared with participant records for labeling purposes.

## 5. Edge-Based System Architecture

**Figure 2:**
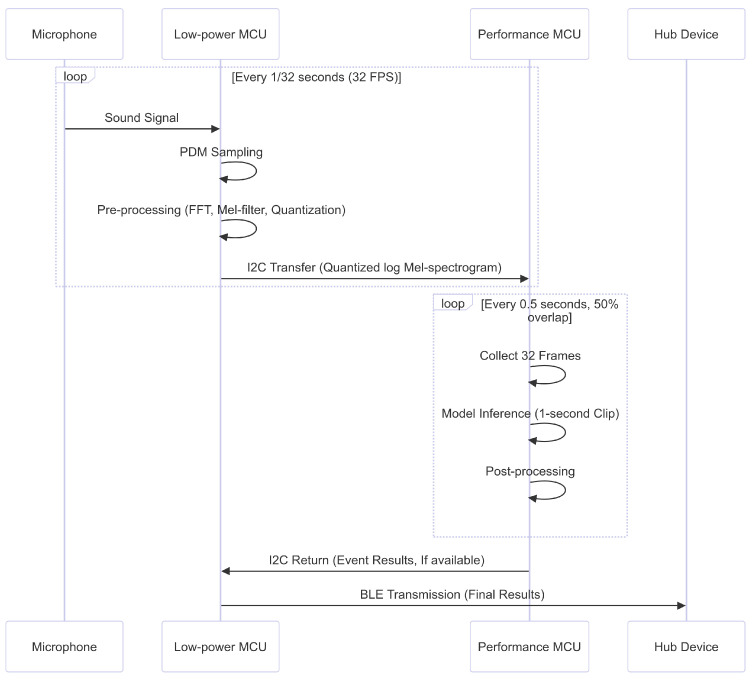
Edge-based system architecture overview.

The edge nodes are designed to operate wirelessly, eliminating the need for power or network cables. They function on an event-driven basis, activating and operating only when there is a substantial noise level, and remaining in standby mode during quiet periods to conserve power. This design enables effortless installation in optimal locations for behavior detection, irrespective of the physical layout of the existing living space. The system operation process is shown in Figure 2.

The edge nodes are designed to utilize two types of cores: a low-power MCU and a high-performance MCU. While a high-performance MCU is necessary to generate real-time inference results, continuously operating it would consume significant power, making it unsuitable for prolonged use. To address this, the edge node is designed with a parallel structure that leverages a low-power core for data collection, pre-processing, and communication, and a high-performance core dedicated solely to model inference. The low-power core collects data, performs pre-processing, and conducts a preliminary analysis to determine if the data require inference. If inference is needed, the pre-processed data are sent to the high-performance core via an I2C line. The high-performance core awakens upon an I2C interrupt, uses the model to perform inference on the received data, and carries out online post-processing. If the result needs to be transmitted, it signals the low-power core via an interrupt to indicate that the data are ready. The low-power core then reads these data and transmits them to the hub device via BLE. The specifications of each core are shown in Table 2.

The edge nodes remain in a sleep state until an input exceeds the specified threshold. When sound is detected, the microphone module triggers an interrupt, waking the nodes for data collection. During data acquisition, the edge nodes continuously monitor the energy value of the incoming signal. If the average energy of the frame consistently remains below the threshold, they cease reception and return to sleep mode. The energy calculation formula is illustrated in Equation (1).

(1) $$E_{frame}=\sum_{n=0}^{N-1}\frac{\left|x\right|}{N},N=frame\_length$$

The hub device maintains information about each connected device in a class-based structure. Each device class includes details such as the device type, name, and location. As an inference process is generated for each connected device, the system can efficiently handle data from multiple sources simultaneously. The processed results are subsequently forwarded to a logging process for recording, ensuring the chronological order of events at various locations.

## 6. Sound Sampling and Pre-Processing

A commonly used pre-processing technique for audio data involves generating spectrograms to extract both temporal and spectral characteristics from periodic signals. Spectrograms are typically created by applying a windowing function to waveform data at regular intervals and then performing Fast Fourier Transform (FFT) operations, as described in Equation (2). However, this windowing operation can be computationally intensive, and edge nodes equipped with MCUs may struggle to achieve real-time processing. Therefore, it is essential to implement a structure that performs calculations frame by frame during data reception, with the results being stored in a buffer for subsequent processing.

(2) $$X_{k}=\sum_{n=0}^{N-1}x_{n}e^{-\frac{2\pi i}{n}}kn,k=0,1,\ldots ,N-1$$

**Figure 3:**
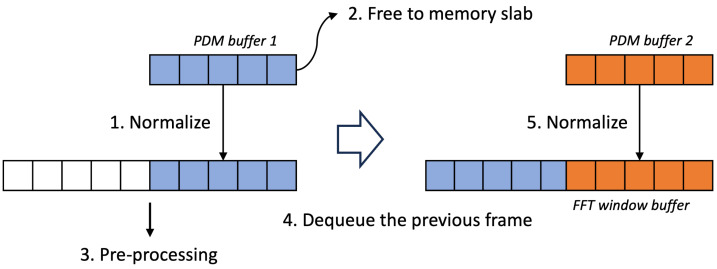
PDM sampling and buffer management for pre-processing.

In our previous research, we verified that the time–frequency resolution of environmental sound data has a relatively small impact on accuracy [42]. Building on this finding, we configured the audio sampling buffer size to match the hop distance between FFT operations, incorporating a 50% window overlap. As shown in Figure 3, we have ensured real-time processing capabilities at the edge node by performing the pre-processing without windowing.

The resulting spectrogram has dimensions of 512×32, which is too large to be directly used as input for deep learning models. Consequently, Mel-spectrograms and MFCCs (Mel-Frequency Cepstral Coefficients) are commonly employed as alternatives to traditional spectrograms. The Mel-spectrogram simplifies the frequency range by applying a Mel-scale filter bank (as shown in Equation (3)) to the spectrogram.

(3) $$m=2595\log_{10}\left(1+\frac{f}{700}\right)$$

In our research, we applied 48 Mel-filters and used a log-transformed 48×32 Mel-spectrogram as the input. In general, 40 Mel-filters are commonly used in speech recognition. This is because 40 filters provide sufficient resolution to represent the frequency range of 80 Hz to 8000 Hz, which contains the key features of speech signals. However, unlike speech signals, noise signals can have characteristics across all frequency bands and may carry significant information even in regions that humans cannot hear. To account for this, a greater number of filters than typically used was employed, and based on the reduction factor through pooling in the model, we utilized $n\times 2^{4}$ filters.

This allowed us to compress the feature data to 6 KB per second. To maintain consistency, we implemented pre-processing operations on the edge node to match the results of the Mel-spectrogram feature extraction function found in the widely used librosa library [43], which is commonly used in sound analysis. While environments like servers typically utilize float-type features directly as input, in edge environments, it is common to employ quantized data to reduce the size and computational load of both the input and model parameters. By quantizing float32 data to int8, the input size is significantly reduced to 1.5 KB per second, with some sacrifice in accuracy. The low-power core then prepares for inference by transmitting these data to the performance core via I2C.

## 7. Training and Deploying Model

### 7.1. Characteristics of Behavioral Sound Data

**Figure 4:**
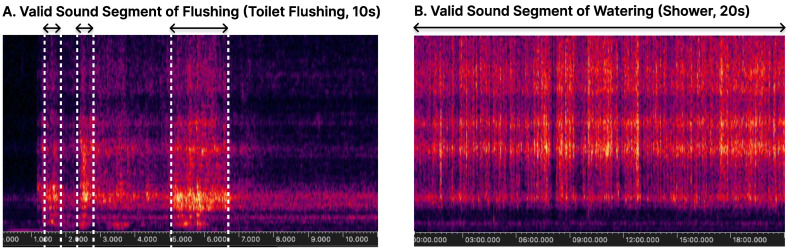
Features of different sound types.

In this study, we utilize a model that infers and identifies sounds based on one-second audio clips. However, sounds occurring in real environments have diverse characteristics, making it challenging to classify all sounds uniformly within a one-second timeframe. For instance, as shown in Figure 4, the sound of flowing water exhibits a consistent and repetitive pattern, whereas sounds like flushing appear briefly over just a few frames. To accurately infer a variety of sound patterns using the same model, it is essential to consider the characteristics of each sound throughout various stages, from preparing the training data to post-processing.

We broadly categorized sounds into two types: continuous sounds and short-duration sounds, applying different methods for data collection and processing for each. Continuous sounds can be easily collected by dividing the continuous data into one-second segments without any special processing, allowing us to gather a large amount of data. These sounds also have the characteristic of consistently producing the same inference results during the continuous inference process. On the other hand, short-duration sounds, where the entire event occurs within one second, require padding and only allow for the collection of a single piece of training data per event. Therefore, strategies to overcome the limited amount of data are needed. These sounds also typically appear briefly, often only once or twice during the inference process.

Table 3 shows the sounds selected for collection, considering the characteristics of various sounds, the corresponding ADL (Activities of Daily Living) categories, and how they are classified by the model. The data collected based on behavioral events and the classification labels used by the model do not always align. This discrepancy reflects challenges in distinguishing various events using the model in real-world environments or cases where multiple types of sounds occur within a single event. For example, the “water flowing” event is categorized into “watering1” and “watering2” to distinguish between the fast, strong sound of water during a shower and the gentle sound of water during handwashing. Additionally, events like chopping, dropping, and dish clanging have very similar sound characteristics, so they were grouped and trained as a single “hitting” event.

### 7.2. Building Dataset with Real-World Sound Data

Various datasets, such as AudioSet [35], have extensively covered environmental sound data and have been actively utilized in research. However, when a model trained on these datasets is deployed in real-world environments, its performance often decreases due to the effects of domain shift [44]. To mitigate the impact of domain shift, models are typically designed to be more generalizable by using more features as input and increasing model depth. However, models operating in edge environments must produce inference results within a limited time using constrained resources, and they can only utilize quantized data that may have lost fine details. Consequently, the effects of domain shift are particularly pronounced in edge environments. To overcome this, it is necessary to train the base model with data collected using microphones with the same specifications as those installed on the edge nodes, and to adapt the model by collecting data from the actual deployment site before distribution.

In this study, we independently carried out the process from data collection and labeling to model training and deployment to implement a system that operates in real-world environments. We obtained consent from participants to install our system in their actual homes, allowing us to collect sound data and label them for use as a training dataset. The dataset is broadly divided into two categories: data for training the base model and data for specializing the model for the installation site. The data for base model training include some portions of publicly available datasets and were collected from various locations such as laboratories and homes using different types of recording equipment. The dataset for model specialization consists of 1–2 days of data collected using edge nodes in the same environment where the system is to be installed, and it is divided into training and testing data. The model training uses a combined training dataset of base and domain-specific data depending on the location where the edge nodes will be installed.

Domain-specific data collection on edge nodes is conducted in the following manner. When the edge node initially establishes a BLE connection with the hub device, it synchronizes with the hub’s time information and then counts time on its own. The edge node is equipped with an SD card for collecting data for analysis purposes. During data collection mode, the pre-processing and inference processes are not active. Collected data are stored on the SD card in WAV format, with the file name reflecting the time the file was created.

Data recording is triggered similarly to inference mode: recording starts when a sound exceeding a certain threshold is detected and stops when low energy levels are maintained for more than 5 s, using an event-driven approach. Participants are asked to record their actions and corresponding times separately to aid in the labeling process afterward.

Table 4 shows the number of data points in the base dataset and the domain dataset collected from the test environment. The main difference between the base dataset and the domain dataset lies in their diversity. The base dataset includes publicly available datasets, data collected from various types of recording equipment, and data gathered from multiple locations. On the other hand, the domain dataset consists only of sounds recorded by the installed edge devices at a single location and does not include all labeled sounds. For example, it would be hard to collect Microwave sounds in a bathroom environment.

**Figure 5:**
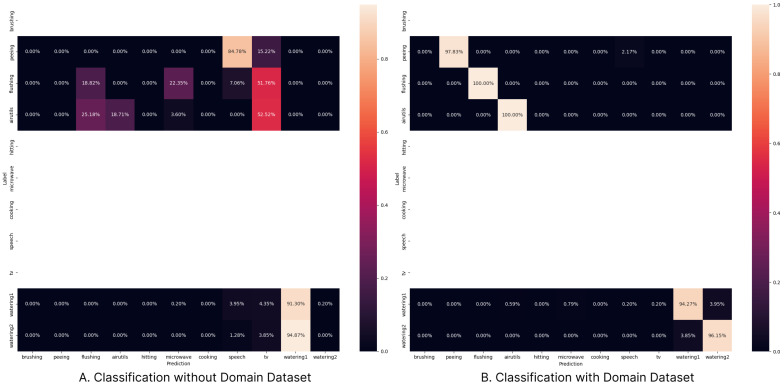
Classification result of Domain1 test dataset.

The role of the base dataset is to train the overall feature extractor of the model, while the domain dataset’s role is to teach the model the characteristics of sounds that occur in a specific environment. The actual training is conducted by combining the base dataset and domain dataset in a 1500:250 ratio. If this process is not followed, it is not possible to properly classify real data, as in Figure 5. The edge device we propose is designed to be permanently installed in a specific location to provide services, where it is more important to accurately classify specific data in a particular domain than to achieve generic performance across all environments. For further work, we plan to conduct research on systematizing the process of tuning the base model into a domain-specific model.

### 7.3. Handling Imbalanced Datasets

As illustrated in Table 4, the original dataset exhibits significant variations in the number of data samples per label. Training models on such imbalanced data can lead to overfitting on specific labels [45]. To mitigate this issue, we resampled the training dataset to contain an equal number of 1500 samples for each label. For labels with fewer than 1500 clips, we applied oversampling techniques, repetitively extracting samples to reach the desired quantity [46]. Similarly, for the domain dataset, resampling was carried based on 250 samples and added to the base dataset. Subsequently, we augmented the resampled dataset by introducing data with some time and frequency information randomly removed [47]. This augmentation process generated two augmented data samples for each clip. As a result, we obtained a total of over 4500 training data samples for each label, ensuring robust model training.

### 7.4. Training and Deploying Deep Learning Model for Edge Nodes

**Figure 6:**
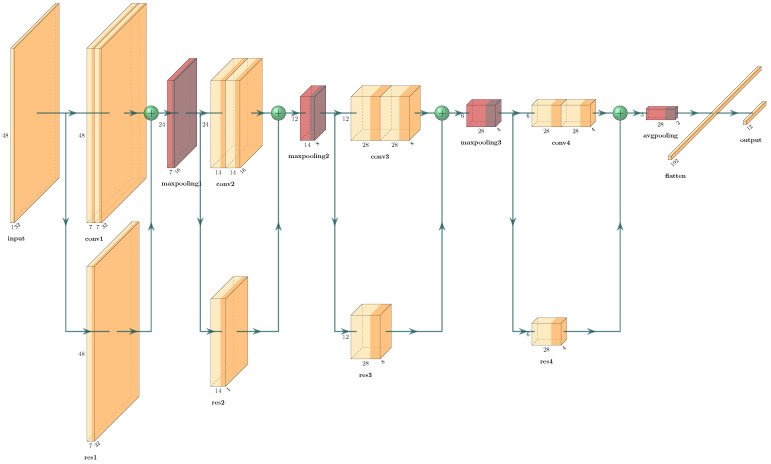
ResNet-based model structure.

To determine the foundational model for the system, we tested several model architectures to find the optimal balance between accuracy and inference performance. The models we considered for testing included a 1D CNN, 2D CNN, and TCN (Temporal Convolutional Network), LSTM (Long Short-Term Memory), and ResNet base model for handling time-series data. Based on the test results, we selected a ResNet-based model as the foundational model and structured it as shown in Figure 6.

Edge environments often lack the resources necessary for model training. Therefore, model creation is typically carried out in high-performance environments like servers, after which the trained model is deployed at the edge. To optimize the model for the edge environment, optimization processes are integrated into the training phase.

One commonly used optimization technique is quantization, which involves quantizing model parameters to integers. Quantization can reduce the model’s accuracy to some extent, but it significantly improves runtime performance by reducing the amount of floating-point operations. Quantization of model parameters is an essential process for running the model on a microcontroller using TensorFlow Lite for Microcontrollers (TFLM). Additionally, the edge node architecture designed in this study includes a process of exchanging features and results between the low-power core and the performance core. Through quantization, communication data can be reduced by up to one-fourth, enabling smoother communication and real-time operation.

Quantized models are stored in the flatbuffer format and can be loaded and used by the TFLite interpreter running on the performance core. These files contain all the necessary information for inference, including the model’s input/output formats, model weights, and quantization parameters.

## 8. Post-Processing

**Figure 7:**
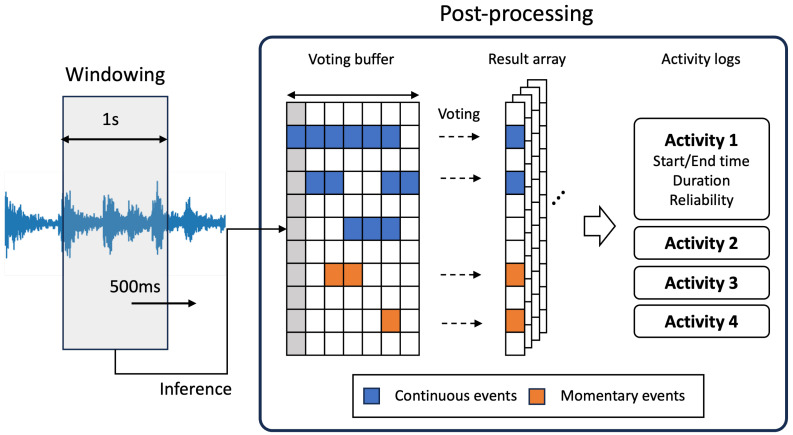
Post-processing overview.

In this study, the model we employed infers 1-second audio clips and provides results. When using such a model for continuous analysis of ongoing sounds, it generates a significant amount of event information in a short timeframe. In scenarios where multiple events occur simultaneously or when there is ambient noise, it may exhibit a pattern where various events change rapidly. To measure a user’s ADL, obtaining reliable information about the action, including the type of action, time of occurrence, and duration, is crucial. However, when multiple labels change randomly, it becomes challenging to obtain accurate action and timing information. To address this issue, we applied a voting-based post-processing method, as illustrated in Figure 7, to refine the action data. Additionally, based on this, we transformed the occurring actions into event logs to facilitate higher-level analysis.

Voting, a method commonly used in ensemble models, combines results from multiple models to produce a single outcome when making inferences on the same data. We use it to ensure consistent results from a single model by assuming that consecutive input data frames share the same label. This assumption is grounded in our analysis of recorded data, which revealed that instances of actions changing multiple times within a 1-second window were extremely rare, except for brief events like drops or door slams. Furthermore, a short event’s occurrence did not necessarily imply the interruption of a continuous event. For example, during dishwashing, both the sound of water flowing and the sound of dishes clanging can occur simultaneously without one interrupting the other.

Traditional voting methods can be categorized as hard voting and soft voting. In hard voting, the final decision is based on the most frequent result from multiple inferences. However, this approach can potentially lose information from events with slightly lower confidence, especially when multiple events occur simultaneously. On the other hand, soft voting computes the average of all inference results, which ensures that events with lower confidence still contribute to the final decision. However, soft voting may not effectively handle short-duration events due to its reliance on the overall average.

To overcome the drawbacks of both methods, we independently assess the occurrence of each event without linking it to other labels. This approach allows us to provide confidence values for additional analysis. Moreover, we classify events into continuous and momentary categories and process them differently to prevent the loss of information from short-duration events.

In this section, we refer to the results inferred from a single post-processing buffer as an “event”, and we represent the abstracted behavioral data that we aim to obtain through post-processing as an “activity”.

We segmented the continuous audio signal into 1-second frames with a 50% overlap, performing inference every 0.5 s. These frames were then grouped into sets of seven frames (equivalent to 3.5 s) and subjected to windowing. Within each window, we counted the number of inferences with probabilities exceeding a specific threshold for each label. For continuous events, if the count exceeded half of the window size, we deemed the event to have occurred. The confidence score was computed by averaging the probabilities within the subsequent processing window. In the case of momentary events, we assumed they had occurred if the inference result exceeded the threshold value. The confidence score was determined based on the number of detections within the frame, resulting in an average probability.

This process aims to improve the reliability of continuous events and preserve the occurrence of momentary events. Based on this, we transform the inference results into an activity log. Activity information includes start and end times, duration, occurrence location, and confidence. The occurrence location refers to the spatial unit where the data are transmitted from the installed edge node, while confidence represents the average confidence of the continuous events that compose the activity. Each event has a tolerance value for each label, which determines the time interval allowed to consider consecutive identical events as a single activity. For continuous activity, we set tolerance to 5 s or more to prevent sustained activity from being interrupted by short activities. For momentary activity, we set it to be shorter, typically 3 to 5 counts, to report it as they occur.

When a sequence of events exceeds the tolerance value, indicating the end of an activity, we record the end time and calculate the duration and confidence of the activity. Subsequently, if necessary, events with excessively short duration or low confidence can be further filtered. The detailed post-processing procedure is outlined in Algorithm 1.
**Algorithm 1** Post-processing 1:**repeat** 2:    store prediction results in post-processing buffer 3:**until** buffer is full 4:▹ Process for smoothing events and enhancing accuracy for continuous activity 5:count← count the prediction results for each label >threshold ▹ filter out the events with low probability 6:**if** label is continuous event **then** 7:    **if** count>buffer_size/2 **then** 8:        set event occurred 9:        confidence← mean of the predictions for the entire buffer10:    **end if**11:**else if** label is instant event **then**12:    set event occurred13:    confidence← mean of the predictions >threshold14:**end if**15:▹ Process for generating abstracted activity logs16:**if** new event is occurred **then**17:    store start_time18:    reset tolerence19:**end if**20:event_count++21:sum_confidence←sum_confidence+confidence22:**if** the continuous event that had occurred previously did not take place this time **then**23:    tolerance−−24:    **if** tolerance<0 **then**25:        store end_time26:        duration←end_time−start_time27:        activity_confidence←sum_confidence/event_count28:        generate the activity log29:    **end if**30:**end if**31:dequeue buffer

## 9. Evaluations

### 9.1. Effects of Applying Resampling, Augmentation, and Quantization

In this study, resampling and augmentation techniques were applied to enhance the model’s classification performance and prevent overfitting. Quantization was also employed as an optimization method to ensure efficient operation in an edge environment. These techniques have a direct impact on the model’s classification performance, and their effects were analyzed prior to model development. For evaluation, we used the ResNet18 model, which serves as the foundation for our approach, as the baseline. This section presents the classification results based on the base dataset, excluding the domain-specific dataset.

Table 5 shows the impact of each process on classification performance. When no techniques are applied, the model shows relatively poor learning outcomes. Applying resampling and augmentation individually results in significant accuracy improvements, with the highest accuracy achieved when all techniques are applied together. Quantization, on the other hand, leads to a slight decrease in classification performance.

### 9.2. Model Classification and Runtime Performance

To address training data variations due to varying action event durations, we used both sampling and augmentation techniques. Table 6 displays results, comparing scenarios with only sampling, only augmentation, and both techniques. We evaluated various models and performed performance assessment with int8 quantization using TensorFlow Lite’s tool. LSTM performance was assessed before quantization, since it lacks quantization support.

The experimental results showed that the LeNet5 model, with its relatively high proportion of Dense layers, achieved the highest accuracy. The ResNet series models also demonstrated strong performance, comparable to other models, while utilizing significantly fewer parameters and consuming less memory. However, despite the lower number of parameters, the actual execution speed did not improve as much as anticipated. This may be attributed to the higher proportion of CNN operations and the deeper layers compared to other models.

The significant difference in memory usage is due to the Dense layers in the model, which have a large number of parameters relative to the execution time. We adjusted the model, while maintaining its structure for comparative analysis, to achieve affordable accuracy while reducing the execution speed. Memory usage can be seen as a characteristic of the model’s structure and does not always correlate with execution time.

**Figure 8:**
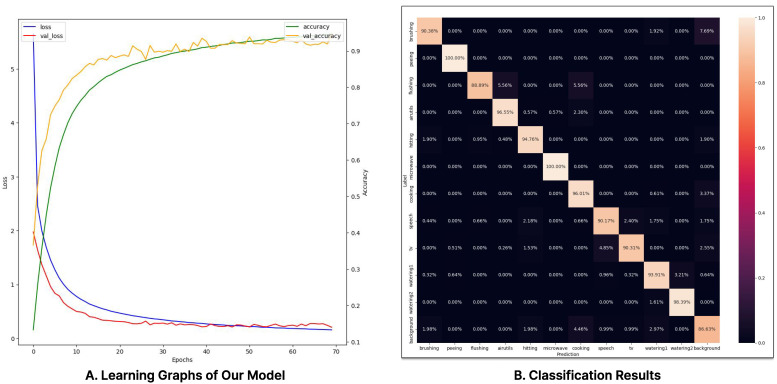
Learning graph and classification results.

The TCN model exhibited some weaknesses in processing Mel-spectrograms. On the other hand, the LSTM model achieved the highest classification performance but could not be deployed on edge nodes, as TensorFlow Lite (TfLite) does not support quantization for LSTM models. Although our model did not achieve the best classification performance, it was confirmed to be suitable for deployment on edge nodes due to its minimal memory usage and its ability to meet the 500 ms time limit required for real-time inference, even with a 50% overlap. The model training graph and classification results are shown in Figure 8. The phenomenon of the validation accuracy initially surpassing the training accuracy is likely due to the application of augmentation only to the training dataset.

### 9.3. Post-Processing

#### 9.3.1. Improving Continuous Event Classification via Voting

The post-processing carried out in this study serves the purpose of enhancing the accuracy and duration estimation of events, even in cases where brief misclassifications occur during long events or when new events emerge. To validate this, we sequentially inferred files longer than 10 s from the test dataset to assess their accuracy. Given that the test dataset comprises data consistently labeled under the same category, effective post-processing should rectify errors in the inference results, resulting in improved accuracy.

We conducted experiments on 5364 clips obtained through pre-processing with a 75% overlap, resulting in a total of 137 files longer than 10 s. As the post-processing group processed seven clips, the number of results after post-processing is 4470.

Table 7 shows the performance improvement for classifying continuous events after voting. Due to imbalances in the test data caused by variations in file length for each event, accuracy measurements utilize the overall average of label-specific classification accuracy. The overall low F1 score is attributed to the fact that only data longer than a certain length were selected, resulting in a lack of data for short events.

The experimental results showcased enhancements in both accuracy and F1 score, all while maintaining the same loss value. These findings suggest that misclassified labels in the inference results of continuous data were effectively rectified. Consequently, it has been verified that post-processing can improve the reliability of continuous events.

#### 9.3.2. Extracting Activity Logs from Noisy Data

**Figure 9:**
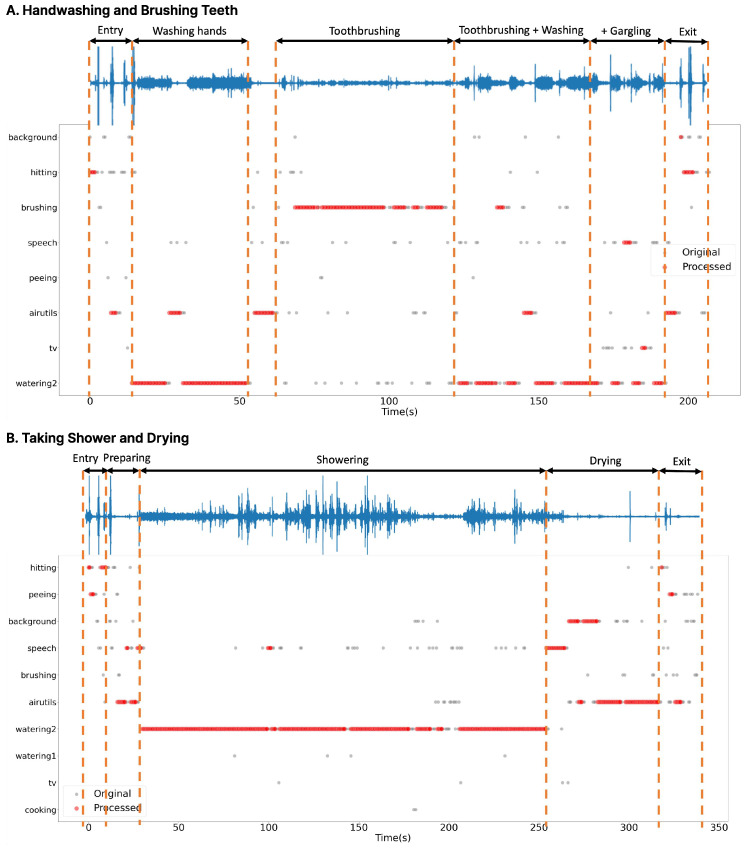
Post-processing noisy data.

Figure 9 and Table 8 depict the initial inference results alongside the results after implementing post-processing on noisy data obtained from a real-world environment. In the pre-processing phase, we presented outcomes for most instances where the count surpassed the threshold within the window, omitting event types and confidence evaluations.

As depicted in Figure 9, the initial inference results show a multitude of unrelated scenarios. However, after applying the post-processing step, the results are predominantly organized based on labels associated with specific actions. Although some misclassified labels may still emerge in noisy environments, they can be further filtered during the log generation process.

Activity log generation involves considering various factors, including event type, confidence level, installation location, and duration, based on the post-processing results. Initially, activities with low confidence can be excluded using the final confidence value. For continuous activities, a tolerance period is set to 5 s for situations where the sound briefly stops or short events occur without interrupting the ongoing event. Additionally, in cases where the installation location is identified as a bathroom, labels such as “eating” or “TV” cannot occur and will be automatically excluded from consideration.

The resulting log data are presented in Table 8. The “Airutils” label represents the sound of a ventilation fan operating in the bathroom, which is detected when no other sounds are present. The “Hitting” label is classified as a short event and is processed separately; thus, it can be observed that it appears briefly between other continuous events. This indicates that even during ongoing continuous events, short sounds, such as the clinking of a cup while brushing teeth or the closing of a door, can be effectively detected. It is evident that the generated logs closely resemble real events. Sounds such as “gargling”, which are not included in existing classification categories, were classified as “speech” due to their similarity to human voices. This highlights the importance of incorporating an outlier detection mechanism to eliminate labels that do not pertain to the intended classification targets in real-world settings.

### 9.4. Predictability and Stability of the System

#### Predictability of the Pre-Processing Process in the Edge Node

The edge node returns a PDM buffer containing 512 samples. Each time this buffer is received, it is combined with the previous buffer for pre-processing before being transmitted to the hub device. Therefore, to ensure real-time performance, pre-processing and transmission must be completed before the next PDM buffer is returned. Since the data collected by the edge node consist of 16,000 samples per second, the processing must be completed within 32 milliseconds to ensure reliable real-time execution. To verify this, we measured the time it takes from when the input PDM buffer is received, which indicates the collection of one frame of data, to the completion of pre-processing and data transmission. Table 9 presents the average processing speed and standard deviation from 100 executions.

**Figure 10:**
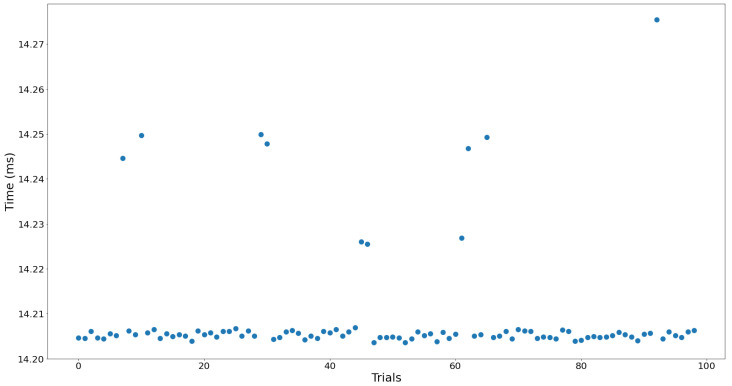
Elapsed time of pre-processing in the edge node.

Figure 10 shows the actual execution time for 100 iterations of pre-processing and transmission on the edge nodes. The experimental results in Table 9 show that the pre-processing and transmission process takes an average of 14.21 ms with a deviation of 13 ns, confirming stable real-time operation. Here, the average time spent on data transmission was 0.4 ms, which had minimal impact on the overall execution time. This is attributed to the architecture of the nRF5340 MCU, where the Application core only stores the generated packets from pre-processing results in shared memory, while the actual transmission is performed in parallel by the Network core.

## 10. Conclusions

In this study, we have developed a real-time system for analyzing residents’ activities based on noise sound information generated in residential areas. To achieve this, we implemented the proposed system in real-life living environments, collected a dataset for training purposes, and built a baseline model that achieved an accuracy over 90% for inference. Additionally, we introduced a post-processing method to enhance event classification, resulting in a classification accuracy of up to 96% for sustained events. Based on this, we have proposed a method for generating log data for high-level analysis.

In our research, we tailored the system to adapt to the installation environment based on field-collected data to enhance model performance. In future research, to ensure system versatility, we aim to implement a system that can adapt to varying environments using techniques like transfer learning. Moreover, since it is impractical to classify all sounds in a given space, we plan to incorporate outlier detection technology to filter out sounds beyond the scope of classification.

For high-level analysis, we successfully abstracted inference results to generate log data. In future research, we intend to integrate these data with activity information obtained from sources other than sound to accurately detect activities and identify abnormal behaviors. Ultimately, our goal is to implement an AAL system that leverages detected behaviors and information on anomalous behavior to provide tailored recommendations for improving the ability to perform ADL.

## Figures and Tables

**Table 1:** Summary of related works.

Method	Ref.	Data	Contribution
Smartphone-based HAR	[15]	Accel, Gyro, Lin Acc (H-Activity, MHEALTH, UCI-HAR)	CNN-LSTM with self-attention for 99.93% accuracy on H-Activity.
	[16]	Accel (UCI-HAR)	4-layer CNN-LSTM, +2.24% accuracy.
	[17]	Accel (5-class data)	CNN, 96.4% accuracy, 8-bit quantization for speed.
Wearable HAR	[18]	3D-ACC, ECG, PPG (PPG-DaLiA)	Feature fusion, +3.00% F1 score.
	[19]	HR, Motion data	MINIROCKET for feature extraction, superior results at lower cost.
	[20]	Wearable sensor (6 activities)	Lightweight DL model, optimized for edge devices.
IoT-Based Monitoring	[21]	Env Sensors (Temp, Humidity, Flame)	IoT platform for real-time ADL monitoring.
	[11]	MEMS Accel, Temp/Hum sensors	Real-time quarantine monitoring with alerts.
Environmental Sensor HAR	[7]	Air quality sensors	ADL detection using air composition.
Video/Multimodal HAR	[22]	Skeleton, RGB (Kinect)	RGB and skeleton fusion for activity recognition.
Thermal and IoT Activity Recog.	[12]	RGB, Thermal (IoT)	3D thermal model for appliance activity recognition.
UWB Radar-based HAR	[23]	UWB Radar (15 ADLs)	80% accuracy with UWB radar for ADL recognition.
Audio-based HAR	[24]	Audio embeddings (Online videos)	Audio-based ADL recognition, 64.2% (Top-1) accuracy.
Edge-based Sound Class.	[25]	Mel spectrograms (ESC-50, Office)	Tiny Transformer, 99.85% fewer params than CNN.
	[26]	Raw audio (ESC-10, ESC-50, UrbanSound8K)	ACDNet, 97.22% size reduction with strong accuracy.
Cyber–Physical Edge Analytics	[27]	Env Sound (6 office sounds)	Ensemble DNN for event detection with low latency.
Edge-based HAR	[20]	Wearable sensor (6 activities)	Lightweight model, outperforming existing DL techniques.

**Table 2:** Hardware specification.

	Low-Power MCU	Performance MCU
Processor	Arm Cortex-M33	Arm Cortex-M7
Clock	64 MHz	550 MHz
Memory	512 KB	1 MB
Power source	3.7 V Li-ion Battery	

**Table 3:** List of sound events recorded for ADL analysis.

Location	ADL	Events	Labels
Kitchen	Feeding/Cooking	Dish Clanging	Hitting
		Boiling/Frying	Cooking
		Microwave	Microwave
		Chopping	Hitting
		Smoke Extractor	Airutils
Bathroom	Bathing	Water Flowing	Watering1/Watering2
		Toothbrushing	Brushing
	Toileting	Flushing	Flushing
		Peeing	Peeing
Everywhere	Cleaning	Vacuuming	Airutils
	Communication	Speech	Speech
		TV	TV
	Transferring	Door	Hitting
	Anomalous Events	Drop	Hitting

**Table 4:** Data counts for base and example domain datasets.

Label	Base	Domain1	Domain2	Domain3
airutils	3970	347	637	423
brushing	1560		394	53
cooking	3070			1032
flushing	660	212	80	
hitting	1290			324
microwave	1930			832
peeing	920	230	128	
speech	4220			596
tv	5920			1253
watering1	3060	1265		
watering2	1840	195	1978	938

**Table 5:** Performance depending on dataset processing.

Process	Accuracy	Loss	F1
None	0.843	0.475	0.829
Resampling	0.937	0.183	0.938
Augmentation	0.94	0.172	0.939
Res+Aug	0.950	0.163	0.951
Quantization	0.942	0.190	0.941

**Table 6:** Model classification and runtime performance on performance core.

Model	Accuracy	Loss	F1 Score	Parameters	Memory (Bytes)	Runtime (ms)
**Our Model**	**0.932**	**0.216**	**0.934**	**31,386**	**47,936**	**484**
LeNet5	0.943	0.176	0.949	303,762	311,272	740
VGG13	0.915	0.257	0.911	246,980	269,336	731
ResNet18	0.942	0.190	0.941	46,854	69,872	841
TCN	0.867	0.334	0.885	245,740	396,736	1451
LSTM (unquantized)	0.950	0.158	0.945	141,580	Quantization Unavailable	

**Table 7:** Performance with post-processing.

	Acc	Loss	F1
Original	0.930	0.231	0.744
Processed	0.963	0.235	0.776

**Table 8:** Generated logs.

**A. Handwashing and Brushing Teeth**				
**Start (s)**	**End (s)**	**Label**	**Duration (s)**	**Reliability**
0.00	8.00	hitting	8.00	0.80
13.50	15.25	airutils	1.75	0.64
7.25	26.25	watering2	19.00	0.97
28.25	30.75	airutils	2.50	0.73
30.25	35.75	hitting	5.50	0.83
34.50	59.00	brushing	24.50	0.79
68.25	69.25	brushing	1.00	0.68
72.75	74.00	airutils	1.25	0.65
73.25	75.25	hitting	2.00	0.38
89.50	90.50	speech	1.00	0.70
96.50	98.00	airutils	1.50	0.68
97.75	102.25	hitting	4.50	0.73
62.00	95.75	watering2	33.75	0.87
**B. Taking Shower and Drying**				
**Start (s)**	**End (s)**	**Label**	**Duration (s)**	**Reliability**
8.25	13.25	airutils	5.00	0.67
0.00	14.50	hitting	14.50	0.70
13.75	14.75	speech	1.00	0.70
15.00	127.00	watering2	112.00	0.91
127.25	132.25	speech	5.00	0.80
133.50	141.25	background	7.75	0.74
161.50	162.25	peeing	0.75	0.73
141.50	164.50	airutils	23.00	0.73
148.25	161.00	hitting	12.75	0.70

**Table 9:** Average time and standard deviation for pre-processing in the edge node.

Average	Deviation
14.209 ms	13.129 ns

## Data Availability

The datasets presented in this article are not readily available because the data provider did not grant permission for sharing. Requests to access the datasets should be directed to authors.
